# Risk factors and predictive value of perioperative neurocognitive disorders in elderly patients with gastrointestinal tumors

**DOI:** 10.1186/s12871-021-01405-7

**Published:** 2021-07-19

**Authors:** Yong-Li Li, Hui-Fan Huang, Yuan Le

**Affiliations:** 1 Department of Anesthesiology, the Third Xiangya Hospital, Central South University, No.138, Tongzipo Road, Yuelu District, Changsha, 410013 Hunan China; 2 Hunan Province Key Laboratory of Brain Homeostasis, The Third Xiangya Hospital, Central South University, No.138, Tongzipo Road, Yuelu District, Changsha, 410013 Hunan China

**Keywords:** Perioperative neurocognitive disorders, Postoperative cognitive dysfunction, Gastrointestinal tumors, Risk factors, Elderly

## Abstract

**Background:**

This study aims to investigate the risk factors of perioperative neurocognitive disorders (PNDs) mainly including postoperative cognitive dysfunction (POCD) in elderly patients with gastrointestinal tumors, and evaluate its predictive value.

**Methods:**

A total of 222 eligible elderly patients (≥65 years) scheduled for elective gastroenterectomy under general anesthesia were enrolled. The cognitive function assessment was carried out 1 day before surgery and 7 days after surgery. Receiver operating characteristic curve analysis was performed to evaluate the predictive value of risk factors for early POCD. The risk factors for POCD were analyzed using univariate and multivariate logistic regression model.

**Results:**

Of all the 222 enrolled patients, 91 (41.0%) developed early POCD and 40 (18.0%) were identified as major POCD within 7 days after the surgery. Visual analogue score (VAS, 1st day, resting) ≥4 (OR = 7.618[3.231–17.962], *P* < 0.001) and alcohol exposure (OR = 2.398[1.174–4.900], *P* = 0.016) were independent risk factors for early POCD. VAS score (1st, resting) ≥4 (OR = 13.823[4.779–39.981], *P* < 0.001), preoperative white blood cell (WBC) levels ≥10 × 10*9/L (OR = 5.548[1.128–26.221], *P* = 0.035), blood loss ≥500 ml (OR = 3.317[1.094–10.059], *P* = 0.034), history of hypertension (OR = 3.046[1.267–7.322], *P* = 0.013), and neutrophil–lymphocyte ratio (NLR) ≥2 (OR = 3.261[1.020–10.419], *P* = 0.046) were independent risk factors for major POCD. Receiver operating characteristic curve analysis indicated that VAS score (1st day, resting) was a significant predictor for major POCD with a cut-off value of 2.68 and an area under the curve of 0.860 (95% confidence interval: 0.801–0.920, *P* < 0.001).

**Conclusions:**

The risk factors for early POCD after gastroenterectomy included high VAS score (1st day, resting) and alcohol exposure. High VAS score, preoperative WBC levels ≥10 × 10*9/L, blood loss ≥500 ml, NLR ≥2, and history of hypertension were independent risk factors for major POCD. Among them, VAS score was one of the important predictors.

**Supplementary Information:**

The online version contains supplementary material available at 10.1186/s12871-021-01405-7.

## Background

Postoperative cognitive disorders (PNDs) is a kind of impairment in cognitive ability which is the most common complication experienced in the postoperative period by these elderly individuals [1–4]. PNDs includes postoperative delirium (POD) and postoperative cognitive dysfunction (POCD) [4]. POD occurs hours to days after surgery and is characterized by cognitive deficits in executive function, memory, and other cognitive domains, with most symptoms resolving in weeks to months [1]. According to previous studies, the incidence of POD in non-cardiac surgery is 13–50% [5]. Delirium is associated with the need for supportive postoperative care, progression to dementia, and increased mortality risk, healthcare costs [6–9]. POCD is cognitive decline performed predominantly in executive function and memory domains of cognition. The incidence of POCD is reported to range from 17 to 43% [10]. Previous clinical studies have identified age, inflammation, and preoperative cognitive disorders as potential risk factors for PNDs [1]. PNDs are measured by a battery of neuropsychological tests, but with a range of criteria. At present, there are still many controversies about the pathological mechanism and treatment of PNDs. Therefore, identifying and avoiding its risk factors may be an effective strategy.

Gastrointestinal tumors mainly include gastric cancer and colorectal cancer. Gastric carcinoma (GC) is the fourth most common malignant tumor and remains the second most deadly cancer of all malignancies worldwide [11–13]. Colorectal cancer (CRC) is the world’s fourth cause of death cancer with almost 900,000 deaths annually [14]. The incidence of gastrointestinal tumors varies geographically with the highest rates seen in the most developed countries. Gastrointestinal tumors have caused a serious global financial medical burden [15–17]. In epidemiological studies, smoking, excessive alcohol intake, obesity, red and processed meat intake, type 2 diabetes and increasing age have shown strong associations with disease incidence. Both hereditary and environmental play an important part in the development of disease [11, 14]. Treatments include endoscopic and surgical therapy, radiotherapy and systemic therapy, and palliative chemotherapy, targeted therapy, and immunotherapy [11–14]. The elderly patients are vulnerable to PNDs after surgery. However, the risk factors for PNDs in patients with gastrointestinal tumors remain unclear. This study aimed to reveal the potential risk factors for PNDs in elderly patients with gastrointestinal tumors and evaluate their predictive value.

## Methods

### Patients

The present study protocol was approved by the Ethics Committee of the Third Xiangya Hospital (ID:21011). All methods were performed in accordance with the relevant guidelines and regulations. Elderly patients with gastrointestinal tumors (aged ≥65 years) who were scheduled for selective Gastroenterectomy under general anesthesia in our hospital from January 2018 to June 2020 were enrolled in this study. Inclusion criteria were: (1) Preoperative Mini-Mental State Examination (MMSE) scores ≥24 points; (2) aged ≥65 years; (3) patient was scheduled for elective Gastroenterectomy under general anesthesia. In addition, the exclusion criteria were as follows: (1) history of severe neurological or psychiatric disease; (2) history of tranquilizers or antidepressants medication; (3) serious audio-visual impairments that affected assessments; (4) patients diagnosed with delirium by using CAM before surgery.

### Data collection

The following data were collected: (1) demographics and clinical baseline data, including age, gender, education, and body mass index (BMI), American Society of Anesthesiologists grade (ASA), MMSE score, smoking or drinking habits, medical history; (2) main clinicopathological parameters, including the type of surgery, operation time and estimated blood loss, perioperative insulation, VAS score (1st, 2nd, 7th day after surgery, resting and activity), EQ-5D score (7th, 30th day after surgery), grip strength, transverse abdominis plane block (TAP), intensive care unit (ICU) therapy, pharmacotherapy (Non-steroidal anti-inflammatory drugs, Dexmedetomidine, etc.); (3) laboratory tests, including the blood cell analysis which was carried out 1 day before surgery and 1 day after surgery, albumin (Alb), creatinine levels, preoperative serum potassium levels (K^+^ levels).

### Cognitive function measurement

The cognitive function assessment was carried at baseline (1 day before the surgery) and at day 7 after the surgery, independently by two experienced anesthesia nurses who were blinded to this protocol. The two anesthesia nurses were professionally trained, and performed Kappa tests for the diagnostic results of POD and POCD before recruiting patients. Delirium was assessed once on preoperatively 1 day and twice daily at 10 am and 6 pm from postoperatively day1 to day 7 using the CAM scale. The battery of neuropsychological tests consisted of mini-mental state examination (MMSE) and the confusion assessment method (CAM). As described by previous Studies, POCD was diagnosed when the MMSE score was lower than 1 standard deviation (SD) compared with the baseline score. A decrease of more than 2 SD in MMSE score was diagnosed as severe cognitive impairment. A decrease in MMSE score of 1 to < 2 SD indicated mild POCD [2, 18, 19].

### Statistical analysis

Data analysis was performed using SPSS 25.0 (SPSS Inc., Chicago, IL). Categorical and continuous data were expressed as number (with percentage, n%) and mean (with standard deviation). Intergroup rates were compared using the chi-square test or Fisher’s test. Relative risk was represented by odds ratio (OR) and its 95% confidence interval (CI). Student’s t test or analysis of variance were used to compare the continuous data between groups according to the data. Repeated measure analysis of variance was used to statistically analyze the VAS scores of the two groups at each period after surgery. The risk factors were analyzed by univariate and multivariate binary Logistic regression analysis. Univariate Logistic regression analysis was used to select independent variables, and relevant factors of *P* value ≤0.15 in univariate analysis were included in the multivariate binary Logistic regression model to analyze the risk factors of perioperative cognitive dysfunction in elderly patients with gastrointestinal tumor. Enter method was used, and *P* value < 0.05 was considered statistically significant. Receiver-operating characteristic curve (ROC) analysis was conducted to assess the predictive value of risk factors for early POCD. The cut-off point value is calculated according to the maximum value of Youden’s index. Statistical significance was set as bilateral *P* value < 0.05. For a small amount of missing data, using the method of average fill for statistical analysis.

## Results

### Demographics and clinical baseline data

A total of 222 elderly patients (≥65 years old) with gastrointestinal tumors were included in the final analysis. 91 of them were identified as early POCD with an incidence of 40.99% (91 of 222) and 40 cases were diagnosed with severe POCD with an incidence of 18.02% (40 of 222). Only 3 of them were diagnosed as POD with an incidence of 1.35% (3 of 222). As the incidence of POD was too low and the existing sample size was too small, the risk factors of POD were not further analyzed. The demographic and clinical characteristics of the patients with or without POCD were summarized in Table 1. Patients with chronic smoking habits (*P* = 0.016) or alcohol consumption (*P* = 0.028) were more likely to suffer from early POCD. No significant differences were observed between POCD and non-POCD groups concerning age, gender, BMI, ASA status, education levels, pre-MMSE score, and medical history (*P* > 0.05).

**Table 1:** Demographics and Clinical data associated with POCD in elderly patients with gastrointestinal tumors

Item	POCD group (***n*** = 91)	Non-POCD group (***n*** = 131)	***P***-value
**Age (y)**	70.57 ± 4.24	70.91 ± 5.10	0.605
**Male (*n*, %)**	61(67.0%)	95(72.5%)	0.379
**BMI (kg.m**^**−2**^**)**	21.81 ± 2.70	22.47 ± 3.17	0.105
**ASA (*n*, %)**			0.097
**II**	21(23.1%)	39(29.8%)	
**III**	70(76.9%)	89(67.9%)	
**IV**	0(0.0%)	3(2.3%)	
**Education (*n*, %)**			0.758
**Illiteracy**	4(4.4%)	6(4.6%)	
**Elementary school**	42(46.2%)	62(47.3%)	
**High school**	41(45.1%)	53(40.5%)	
**College or higher**	4(4.4%)	10(7.6%)	
**Preoperative MMSE score**	26.98 ± 1.70	26.63 ± 1.81	0.154
**Medical history (*n*, %)**			
**Hypertension**	39(42.9%)	53(40.5)	0.721
**Diabetes**	13(14.3%)	20(15.3%)	0.840
**Cerebrovascular disease**	10(11%)	11(8.4%)	0.516
**Cardiovascular disease**	11(12.1%)	17(13%)	0.844
**Lung disease**	38(41.8)	44(33.6)	0.215
**Chemotherapy**	5(5.5%)	2(1.5%)	0.126
**Smoking (*n*, %)**	40(44%)	37(28.2)	0.016*
**Alcohol consumption (*n*, %)**	24(26.4%)	19(14.5%)	0.028*

Notes: *Abbreviations*: *ASA* American Society of Anesthesiologists, *BMI* Body mass index, *MMSE* Mini-mental State Examination, *POCD* Postoperative cognitive dysfunction

* P < 0.05

### Main clinical data and laboratory tests

**Fig. 1:**
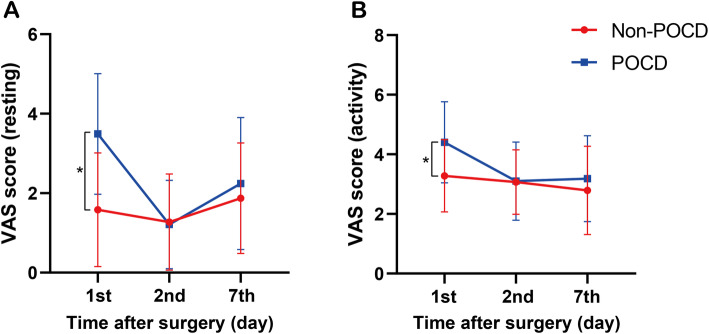
**A**-**B**: Postoperative VAS score and POCD. VAS: visual analogue score; POCD: postoperative cognitive dysfunction; * *P* < 0.05.

As shown in Table 2 and Fig. 1, a higher VAS score (*P* < 0.001) was significantly correlated with the development of POCD. Compared with the non-POCD group, POCD patients had lower serum potassium levels before surgery (*P* = 0.045) and EQ-5D scores on 30 days after surgery (*P* = 0.047). No statistical differences were found in the WBC levels, hemoglobin (Hb), albumin, operation time, blood loss, warm treatment, grip strength, pharmacotherapy (Non-steroidal anti-inflammatory drugs, Dexmedetomidine, etc.) between the POCD and non-POCD group (*P* > 0.05). Although there was no statistical difference in postoperative ICU therapy rate between POCD patients and non-POCD patients, the *P* value was very close to the threshold value of 0.05 (*P* = 0.07).

**Table 2:** Main clinical data and laboratory tests associated with POCD in elderly patients with gastrointestinal tumors

Item	POCD group (***n*** = 91)	Non-POCD group (***n*** = 131)	***P***-value
**VAS score**			
**1st day after surgery (resting)**	3.49 ± 1.52	1.58 ± 1.43	0.000*
**1st day after surgery (activity)**	4.40 ± 1.36	3.28 ± 1.21	0.000*
**2nd day after surgery (resting)**	1.21 ± 1.11	1.27 ± 1.21	0.741
**2nd day after surgery (activity)**	3.10 ± 1.31	3.07 ± 1.08	0.812
**7th day after surgery (resting)**	2.24 ± 1.66	1.87 ± 1.39	0.073
**7th day after surgery (activity)**	3.18 ± 1.44	2.79 ± 1.48	0.050
**1st day after surgery (resting) ≥ 4**	28(30.8%)	8(6.1%)	0.000*
**EQ-5D score**			
**Preoperative EQ-5D score**	82.48 ± 9.93	81.30 ± 12.62	0.434
**7th day after surgery**	64.92 ± 15.73	68.87 ± 13.87	0.050
**30th day after surgery**	68.36 ± 20.14	72.99 ± 14.34	0.047*
**Operation time (min)**	237.02 ± 90.06	225.21 ± 74.87	0.289
**Blood loss (ml)**	282.20 ± 40.59	198.63 ± 21.29	0.070
**> 1000 ml**	6(6.6%)	2(1.5%)	0.066
**TAP**	37(40.7%)	48(36.6%)	0.545
**Warm treatment**	67(73.6%)	104(79.4%)	0.315
**Grip strength**	24.08 ± 6.67	25.48 ± 6.98	0.136
**ICU therapy**	11(12.1%)	7(5.3%)	0.070
**Pharmacotherapy**			
**NSAIDs**	11(12.1%)	18(13.7%)	0.719
**Dexmedetomidine**	30(33%)	48(36.6%)	0.573
**Laboratory tests**			
**K**^**+**^ **levels (mmol/L)**	3.38 ± 0.47	3.51 ± 0.48	0.045*
**Pre-WBC levels (× 10**^**9**^**/L)**	6.19 ± 2.14	6.22 ± 1.92	0.900
**Post-WBC levels (×10**^**9**^**/L)**	10.56 ± 3.64	11.43 ± 3.82	0.093
**WBC gap**	4.65 ± 3.07	4.98 ± 3.08	0.432
**Albumin (g/L)**	38.24 ± 4.81	38.11 ± 4.25	0.842
**Hb levels**	116.17 ± 23.76	115.88 ± 23.85	0.930
**NLR ≥ 2**	65(71.4%)	91(69.5%)	0.753

**Notes:** For a small amount of missing data (loss rate < 5%), using the method of average fill for statistical analysis. *Abbreviations*: *VAS* Visual analogue score, *EQ-5D* quality-of-life EuroQol-5 Dimensions, *TAP* transverse abdominis plane block, *ICU* Intensive care unit, *NSAIDS* Non-steroidal anti-inflammatory drugs, *WBC levels* White blood cells levels, *WBC gap* |post-WBC levels - pre-WBC levels|, *Hb* Hemoglobin, *K+ levels* preoperative serum potassium levels, *MMSE* Mini-mental State Examination, *POCD* Postoperative cognitive dysfunction

*P < 0.05

When POCD patients were further stratified into mild and severe patients, as displayed in Table 3, it was found that in addition to insufficient analgesia, history of hypertension, surgical blood loss of ≥500 ml, and patients with NLR ≥2 were more likely to develop into severe cognitive impairment (*P* < 0.05). Patients with severe postoperative cognitive impairment were more likely to be treated in the ICU during this hospitalization (*P* = 0.024). *P* value for preoperative WBC level ≥ 10 × 10*9/L was close to 0.05 (*P* = 0.051).

**Table 3:** Clinicopathological parameters associated with major POCD in elderly patients with gastrointestinal tumors

Item	Major POCD group (***n*** = 40)	Mild POCD group (***n*** = 51)	Non-POCD group (***n*** = 131)	*P*-value
**Hypertension**	23(57.5%)	16(31.4%)	53(40.5%)	0.040*
**ICU therapy**	8(20.0%)	3(5.9%)	7(5.3%)	0.024*
**Pre-WBC levels ≥ 10 (× 10**^**9**^**/L)**	5(12.5%)	2(3.9%)	3(2.3%)	0.051
**VAS (1st day, resting) ≥ 4**	16(40%)	12(23.5%)	8(6.1%)	0.000*
**Blood loss ≥ 500 ml**	9(22.5%)	3(5.9%)	13(9.9%)	0.034*
**NLR ≥ 2**	35(87.5%)	30(58.8%)	91(69.5%)	0.012*

**Notes:** *Abbreviations*: *ICU* Intensive care unit, *Pre-WBC* Preoperative white blood cell, *VAS* Visual analogue score, *NLR* Neutrophil-lymphocyte ratio, *POCD* Postoperative cognitive dysfunction

* P < 0.05

### Risk factors associated with POCD

The risk factors were analyzed by univariate and multivariate binary Logistic regression analysis. Univariate Logistic regression analysis was used to select independent variables, and relevant factors of *P* value ≤0.15 in univariate analysis were included in the multivariate binary Logistic regression model to analyze the risk factors of perioperative cognitive dysfunction in elderly patients with gastrointestinal tumor. Enter method was used, and *P* value < 0.05 was considered statistically significant. Supplementary Table 1 and supplementary table 2 showed the risk factors associated with POCD or major POCD in elderly patients with gastrointestinal tumors by univariate logistic regression analysis. Table 4 and Table 5 summarized the potential independent risk factors between the POCD and non-POCD groups. The results revealed that VAS score (1st day, resting) ≥4 points and alcohol exposure were the independent risk factors associated with POCD. Furthermore, VAS score ≥ 4 points, blood loss > 500 ml, preoperative white blood cell count ≥10 × 10*9/L, NLR ≥2, and history of hypertension were independent risk factors for severe cognitive impairment in elderly patients with gastrointestinal tumors.

**Table 4:** Risk factors associated with POCD in elderly patients with gastrointestinal tumors by multivariate logistic regression analysis

Predictive factors of POCD	OR	95% Confidence interval		***P***-value
		Lower	Upper	
**VAS (1st day, resting) ≥ 4**	7.618	3.231	17.962	0.000*
**Alcohol consumption**	2.398	1.174	4.900	0.016*
**Blood loss ≥ 1000 ml**	5.308	0.998	28.242	0.050

**Notes:** *Abbreviations*: *VAS* Visual analogue score, *POCD* Postoperative cognitive dysfunction, *OR* Odds ratio

* P < 0.05

**Table 5:** Risk factors associated with major POCD in elderly patients with gastrointestinal tumors by multivariate logistic regression analysis

Predictive factors of POCD	OR	95% Confidence interval		***P***-value
		Lower	Upper	
**VAS (1st day, resting) ≥ 4**	13.823	4.779	39.981	0.000*
**Pre-WBC levels ≥ 10**	5.438	1.128	26.221	0.035*
**Blood loss ≥ 500 ml**	3.317	1.094	10.059	0.034*
**Hypertension**	3.046	1.267	7.322	0.013*
**NLR ≥ 2**	3.261	1.020	10.419	0.046*

**Notes:** *Abbreviations*: *VAS* Visual analogue score, *Pre-WBC levels* Preoperative white blood cell, *NLR* Neutrophil-lymphocyte ratio, *POCD* Postoperative cognitive dysfunction, *OR* Odds ratio

*P < 0.05

### VAS score and POCD

**Fig. 2:**
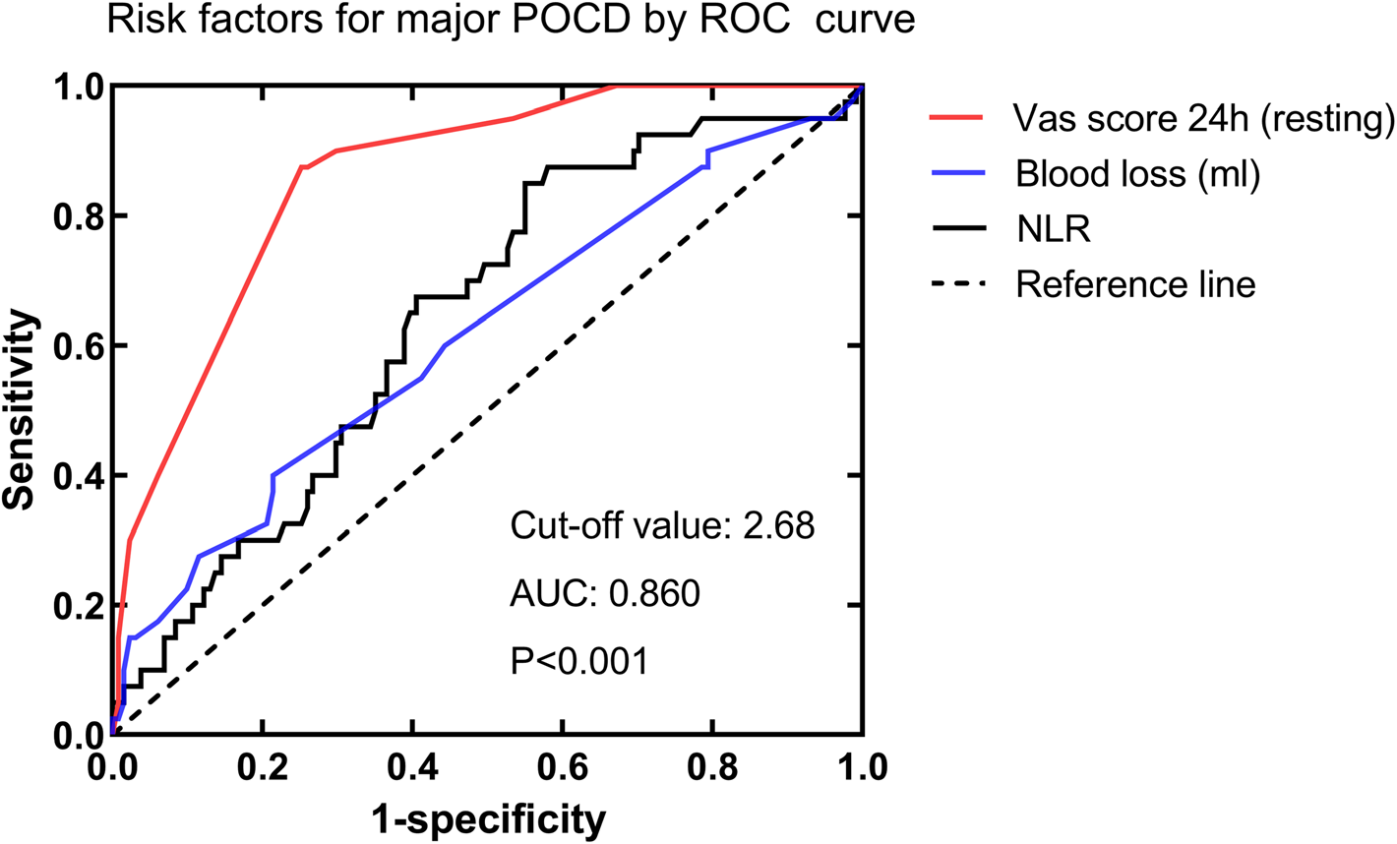
Predictive value of risk factors for early major POCD in elderly patients with gastrointestinal tumors by ROC curve analysis. The area under the curve (AUC) of VAS score for POCD was 0.860, with the cut-off value of 2.68, sensitivity of 87.5% and specificity of 74.8%, respectively (95% CI: 0.801–0.920, *P* < 0.001). ROC: receiver operating characteristic; NLR: neutrophil-lymphocyte ratio; AUC: area under the curve; CI: confidence interval; POCD: postoperative cognitive dysfunction

Receiver-operating characteristic curve analysis was conducted to investigate the predictive value of risk factors for early major POCD. As listed in Fig. 2, the area under the curve of VAS score for POCD was 0.860, with the cut-off value of 2.68, sensitivity of 87.5%, and specificity of 74.8% respectively (95% confidence interval: 0.801–0.920, *P* < 0.001). The ROC analysis of blood loss (AUC: 0.614, *P* = 0.052) and NLR (AUC: 0.644, *P* = 0.047) showed poor predictive performance.

## Discussion

In the present study, we found that the incidence of POCD was calculated to be 41.0 and 18.0% were identified as major POCD within 7 days after the surgery, which was in accordance with the 17 to 43% by Evered L et al. [10] The results from our study indicated high VAS score, hypertension, preoperative WBC levels of ≥10 × 10*9/L, surgical blood loss of ≥500 ml, and patients with NLR ≥2 were independent risk factors for major POCD in elderly patients with gastrointestinal tumors. VAS score was one of the important predictors. Multimodal analgesia and inflammation control may be effective suggestions to minimize the POCD in elderly patients with gastrointestinal tumors. Patients with major POCD were more likely to be treated in the ICU during this hospitalization and related to poor EQ-5D scores on day 30.

PNDs are common postoperative complications after major surgery with unknown exact pathophysiology in the elderly over 65 years old [1, 20]. Previous studies have identified age, lower level of education, inflammation, postoperative pain, serum 25(OH) D level and preoperative cognitive disorders as potential risk factors for PNDs [21–25]. However, it isn’t exactly in accordance with our results. Our results from multivariate logistic analysis did not support the predictive value of age and lower level of education. The relatively small age range and different types of surgery may attribute to the difference. Our study revealed that perioperative pain management and infection control may be an important link to avoid the occurrence of PNDS. Interestingly, Halazun et al. [26] and Yong, R. et al. [27] have also reported the predictive role of preoperative NLR for POCD. However, the cut-off value of NLR for POCD in their results were 5.0 and 2.5, which was quite different from our study (2.0). NLR is calculated from neutrophils and lymphocytes, which is considered as a prognostic factor in patients with numerous diseases, such as lung cancer, colorectal cancer, coronary artery bypass grafting, Alzheimer’s disease and cardiovascular disease [28–32]. As a predictor of disease, NLR is not very specific, but it reveals that inflammation may play an important role in many diseases.

Our results provide a few suggestions to minimize the POCD in elderly patients with gastrointestinal tumors, but we only investigated the risk factors for POCD in the early postoperative period. Previous studies have failed to show an association between cognitive dysfunction lasting months to years after surgery and the anaesthesia itself. At present, relatively few long-term prospective studies have been published, and relevant researches are needed [33–38].

### Limitations

The study has some limitations. First, MMSE, the perioperative cognitive function assessment scale used in this study, has some limitations and its efficacy in screening mild cognitive function is insufficient. Second, the international academic community usually evaluates PND with a combination of neuropsychological tests, but this method is very complex and patients have poor coordination. Therefore, the cognitive assessment tools used in large sample clinical studies need to be further studied. Lastly, multicentre clinical big data and observational studies are needed to determine whether the current risk factors have high predictive value.

## Conclusions

In conclusion, the incidence of POCD is relatively high in elderly patients with gastrointestinal tumors. The risk factors for early POCD after gastroenterectomy included a high resting VAS score on the first day after surgery and alcohol exposure. High VAS score, preoperative WBC levels ≥10 × 10*9, blood loss ≥500 ml, NLR ≥2, and history of hypertension were independent risk factors for major POCD among which VAS score was one of the important predictors.

## Supplementary Information

**Additional file 1: Table S1.** Risk factors associated with POCD in elderly patients with gastrointestinal tumors by univariate logistic regression analysis.

**Additional file 2: Table S2.** Risk factors associated with major POCD in elderly patients with gastrointestinal tumors by univariate logistic regression analysis.

## Data Availability

The datasets generated and analyzed during the current study are available from the corresponding author on reasonable request.

## Abbreviations

PNDs: Perioperative neurocognitive disorders
POCD: Postoperative cognitive dysfunction
POD: Postoperative delirium
