# Supplementary material for: Risk factors and predictive value of perioperative neurocognitive disorders in elderly patients with gastrointestinal tumors
Source: BMC Anesthesiol. 2021 Jul 19;21:193. doi: 10.1186/s12871-021-01405-7 (PMC8287702; doi:10.1186/s12871-021-01405-7)
Supplement: Supplementary file 1 — Additional file 1: Table S1. Risk factors associated with POCD in elderly patients with gastrointestinal tumors by univariate logistic regression analysis. [file 12871_2021_1405_MOESM1_ESM.docx]

Supplementary Table 1: Risk factors associated with POCD in elderly patients with gastrointestinal tumors by univariate logistic regression analysis

| **Predictive factors of POCD** | **OR** | **95% Confidence interval** | | ***P*-value** |
| --- | --- | --- | --- | --- |
|  |  | **Lower** | **Upper** |  |
| **VAS (1^st^ day, resting) ≥4** | 6.833 | 2.943 | 15.866 | 0.000* |
| **Alcohol consumption** | 2.112 | 1.077 | 4.142 | 0.030* |
| **Blood loss ≥1000ml** | 4.553 | 0.898 | 23.088 | 0.067 |

*Notes: Abbreviations: VAS: visual analogue score; POCD: postoperative cognitive dysfunction; OR: odds ratio.*

** P < 0.05.*
