# Supplementary material for: Risk factors and predictive value of perioperative neurocognitive disorders in elderly patients with gastrointestinal tumors
Source: BMC Anesthesiol. 2021 Jul 19;21:193. doi: 10.1186/s12871-021-01405-7 (PMC8287702; doi:10.1186/s12871-021-01405-7)
Supplement: Supplementary file 2 — Additional file 2: Table S2. Risk factors associated with major POCD in elderly patients with gastrointestinal tumors by univariate logistic regression analysis. [file 12871_2021_1405_MOESM2_ESM.docx]

Supplementary Table 2: Risk factors associated with major POCD in elderly patients with gastrointestinal tumors by univariate logistic regression analysis

| **Predictive factors of POCD** | **OR** | **95% Confidence interval** | | ***P*-value** |
| --- | --- | --- | --- | --- |
|  |  | **Lower** | **Upper** |  |
| **VAS (1^st^ day, resting) ≥4** | 10.250 | 3.945 | 26.630 | 0.000* |
| **Pre-WBC levels ≥10** | 6.095 | 1.388 | 26.759 | 0.017* |
| **Blood loss ≥500ml** | 2.635 | 1.032 | 6.729 | 0.043* |
| **Hypertension** | 1.991 | 0.972 | 4.080 | 0.060 |
| **NLR ≥2** | 3.077 | 1.123 | 8.432 | 0.029* |

*Notes: Abbreviations: VAS: visual analogue score; Pre-WBC levels: preoperative white blood cell; NLR:* *neutrophil-lymphocyte ratio; POCD: postoperative cognitive dysfunction; OR: odds ratio.*

** P < 0.05.*
